# Homology modeling and functional annotation of bubaline pregnancy associated glycoprotein 2

**DOI:** 10.1186/2049-1891-3-13

**Published:** 2012-05-31

**Authors:** Bhaskar Ganguly, Shiv Prasad

**Affiliations:** 1 Department of Veterinary Physiology and Biochemistry, College of Veterinary and Animal Sciences, G. B. Pant University of Agriculture and Technology, Pantnagar, PIN: 263145, India; 2 Department of Animal Reproduction, Gynecology and Obstetrics, College of Veterinary and Animal Sciences, G. B. Pant University of Agriculture and Technology, Pantnagar, PIN: 263145, India

**Keywords:** Bubaline, Homology modeling, Pregnancy associated glycoprotein (PAG), Structure, Function

## Abstract

**Background:**

Pregnancy associated glycoproteins form a diverse family of glycoproteins that are variably expressed at different stages of gestation. They are probably involved in immunosuppression of the dam against the feto-maternal placentome. The presence of the products of binucleate cells in maternal circulation has also been correlated with placentogenesis and placental re-modeling. The exact structure and function of the gene product is unknown due to limitations on obtaining purified pregnancy associated glycoprotein preparations.

**Results:**

Our study describes an *in silico* derived 3D model for *bubaline* pregnancy associated glycoprotein 2. Structure-activity features of the protein were characterized, and functional studies predict *bubaline* pregnancy associated glycoprotein 2 as an inducible, extra-cellular, non-essential, N-glycosylated, aspartic pro-endopeptidase that is involved in down-regulation of complement pathway and immunity during pregnancy. The protein is also predicted to be involved in nutritional processes, and apoptotic processes underlying fetal morphogenesis and re-modeling of feto-maternal tissues.

**Conclusion:**

The structural and functional annotation of *bu*PAG2 shall allow the designing of mutants and inhibitors for dissection of the exact physiological role of the protein.

## Background

Pregnancy associated glycoproteins (PAGs) were first isolated in 1982 by Butler and co-workers from the outer epithelial cell layer (chorion/ trophectoderm) of the bovine feto-maternal membranes where they are secreted by binucleate cells [1,2]. Subsequently, PAGs have been isolated from several other species like sheep, goat, buffalo, cat, pig and horse. Presently, more than 100 *PAG* genes are known in ruminants, forming a very diverse family of glycoproteins that are variably expressed at different stages of gestation, starting about 7^th^ day post-fertilization onwards, largely in the pre-placental trophoblast, and post-implantation trophectoderm [3]. Also known as pregnancy specific protein-B (PSPB) or pregnancy specific protein (PSP)-60, these are putatively known to act as immunosuppressants that allow the immunological acceptance of the embryo by the dam. The presence of the products of binucleate cells in maternal circulation has also been correlated with placentogenesis and placental re-modeling [4]. However, the exact structure and function of the gene product remains largely undetermined; limitations on obtaining purified PAG preparations being the major bottleneck. PAGs show high sequence homology as a group, and also to aspartic proteases *viz.* pepsin, cathepsin and chymosin. Given the availability of 3D structures of these homologous proteins, the prediction of PAG structure from its amino acid sequence at high confidence levels is implicit.

In the absence of experimentally determined protein structures, a homology-based model may serve as a good starting point for investigation of sequence-structure-function relationships. Although homology-modeled structures may often not be accurate enough to allow characterization of protein-protein or protein-inhibitor interactions at the atomic level, they can suggest which sequence regions or individual amino acids are essential functional components of the protein. Our study describes the first 3D model for a PAG, using *bubaline* PAG2 (*bu*PAG2) as a candidate, obtained through a combination of several *in silico* modeling approaches. In addition, primary and secondary structure analysis and functional annotation studies were also performed.

## Methods

### Sequence retrieval and analysis

The amino acid sequence of *bu*PAG2 [GenBank: ADO67791.1] was retrieved from GenBank database at NCBI [5]. ProtParam [6] was used to predict physiochemical properties. The parameters computed by ProtParam included the molecular weight, theoretical pI, amino acid composition, atomic composition, extinction coefficient, estimated half-life, instability index, aliphatic index, and grand average of hydropathy (GRAVY).

### 3D modeling of *bu*PAG2

A PSI-BLAST (Position Specific Iterated-Basic Local Alignment Search Tool) [7] search with default parameters was performed against the Protein Data Bank (PDB) to find a suitable template for homology modeling. The template, hence identified, was used for homology modeling using the modeling package MODELLER9v10 [8].

### Model optimization, quality assessment and visualization

Hydrogen addition, and clash reduction was performed in Swiss-Pdb Viewer 4.0.4 [9]. Energy minimization was also performed with *in vacuo* GROMOS96 43B1 parameters set using GROMOS96 implementation in Swiss-Pdb Viewer [10]. The errors in the model were, further, fixed using the tools at What IF Web Interface [11]. For structural evaluation and stereo-chemical analyses, the 3D model was submitted to PDBsum [12]. Overall quality of the structure was determined by ERRAT [13]. Visualization of 3D structures, and superposition, alignment and RMSD determination of query and template structure were performed in YASARA View [14]. For structural alignment, MUSTANG implementation [15] of YASARA View was used.

The glycosylation sites were predicted by using NetOGlyc, NetNGlyc and YinOYang tools, and signal peptide was predicted by SignalP tool, provided by Centre for Biological Sequence Analysis, Technical University of Denmark (CBS DTU) [16,17].

### Protein structure accession number

The final 3D structure of *bu*PAG2 was submitted to the Protein Model Database (PMDB) [18].

### Functional annotation of *bu*PAG2

*Bu*PAG2 was analyzed for the presence of conserved domains based on sequence similarity search with close orthologous family members. For this purpose, three different bioinformatics tools and databases including InterProScan [19], Proteins Families Database (Pfam) [20], and NCBI Conserved Domains Database (NCBI-CDD) [21] were used. InterProScan is a tool that combines different protein signature recognition methods native to the InterPro member databases into one resource with look up of corresponding InterPro and GO annotation. Pfam is a protein family database, including their annotations and multiple sequence alignments generated using hidden Markov models. NCBI-CDD is a protein annotation resource consisting of a collection of well-annotated multiple sequence alignment models for ancient domains and full-length proteins. Additionally, queries were submitted to ProKnow [22] and Kihara Protein Function Prediction (PFP) [23] servers for functional annotation of *bu*PAG2.

Essential proteins of a cellular organism are necessary for survival; information about essentiality of PAG was retrieved from the Database of Essential Genes (DEG) [24]. E-value cut-off of 10^-10^ and a minimum bit score of 100 were used to scan *bu*PAG2 against all essential proteins listed in DEG using BlastP. To check the involvement of PAG into metabolic pathways, KEGG automatic annotation server (KAAS) was used [25].

## Results and discussion

The present study focused on sequence, structural and functional analysis of PAGs using *bu*PAG2 as a model. ProtParam was used to analyze different physiochemical properties from the amino acid sequence. The 367 amino acids long *bu*PAG2 was predicted to have a molecular weight of 40804.7 Daltons and an isoelectric point (pI) of 6.34. An isoelectric point close to 7 indicates a slightly negatively charged protein, and an instability index of 49.21 suggests an unstable protein. The negative GRAVY index of −0.015 is indicative of a hydrophilic and soluble protein.

### Homology modeling of *bu*PAG2

The 3D model of a protein provides us invaluable insights into the structural basis of its function. Homology or comparative modeling is the most common structure prediction method. Numerous online servers and tools are available for homology modeling of proteins. Upon a PSI-BLAST search against the Protein Data Bank (PDB), 3PSG_A was identified as the best template available for the homology modeling of the *bu*PAG2 with 47.59% sequence identity to *bu*PAG2 over 96% query coverage. 3PSG_A is a refined X-ray diffraction model of A-chain of porcine pepsinogen at a resolution of 1.65 Å. The query sequence and template structure were then provided as inputs in MODELLER9v10 to generate the 3D model of *bu*PAG2.

### Energy minimization, quality assessment and visualization

**Figure 1:**
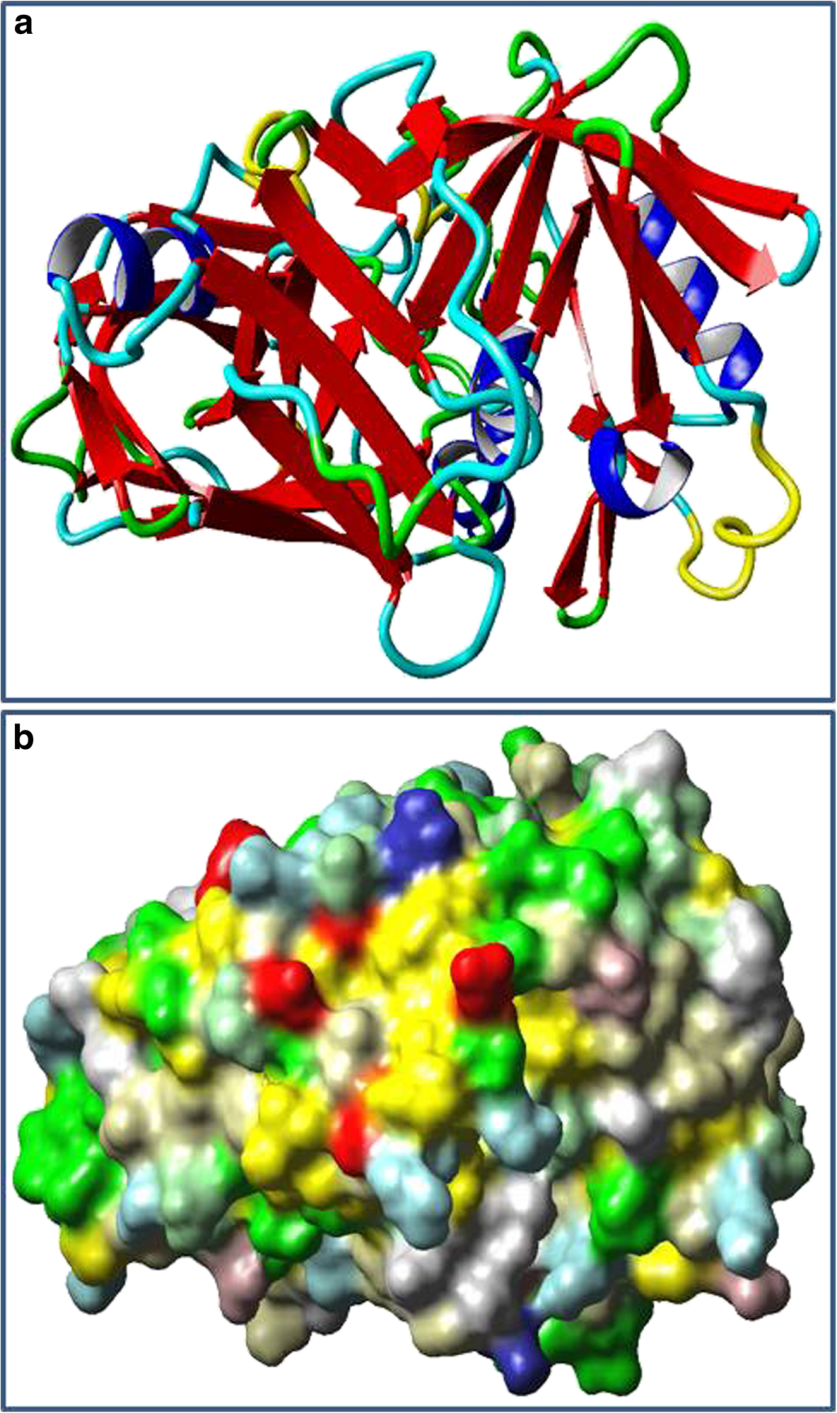
**3D models of*bu*PAG2.** A bi-lobed structure, typical of eukaryotic aspartyl proteases, is evident. **a.** Ribbon model of *bu*PAG2; helices are depicted in blue and sheets in red. **b.** Molecular surface model of *bu*PAG2 colored by ConSurf implementation in YASARA View.

**Figure 2:**
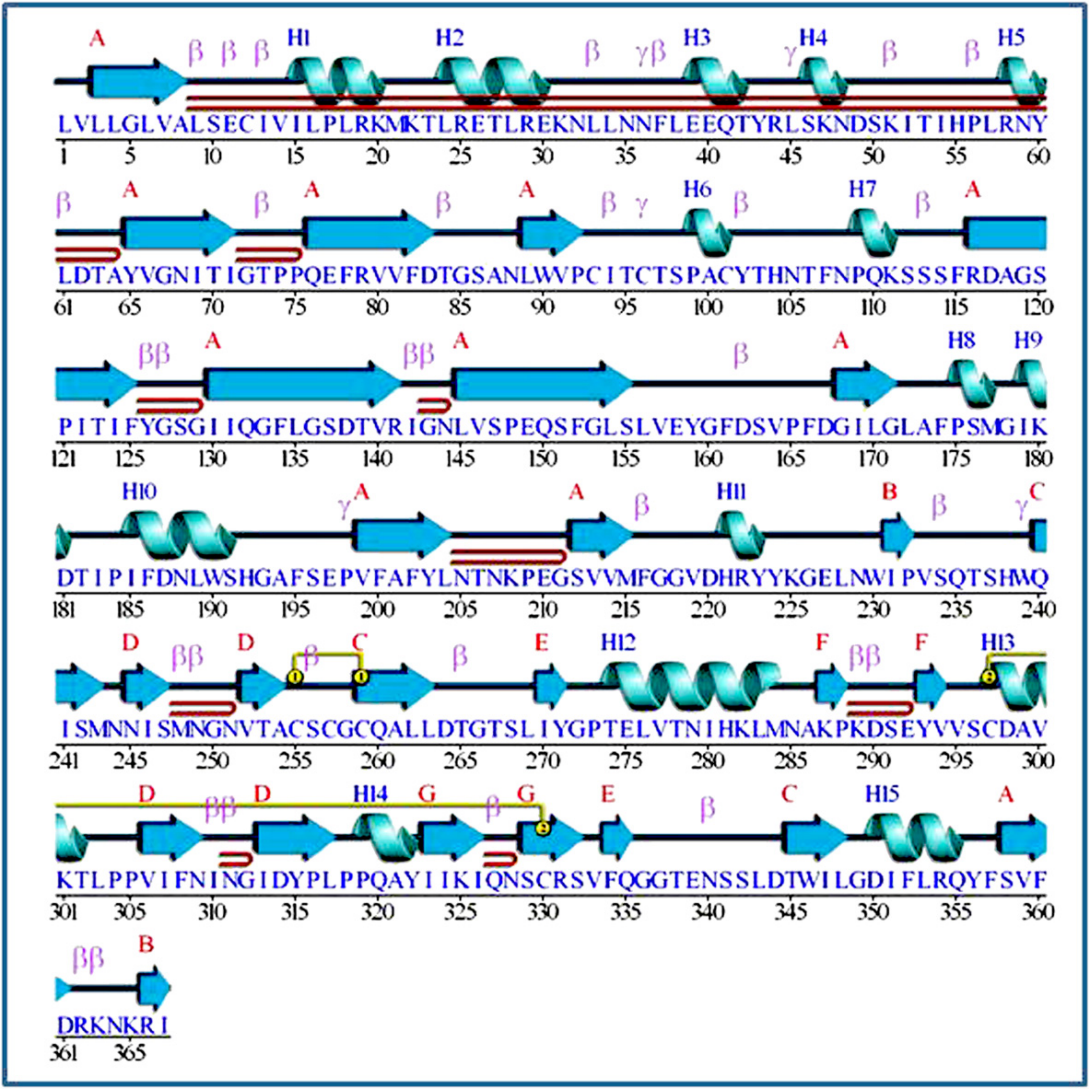
**Predicted secondary structure of*bu*PAG2.** 15 helices and 7 sheets are present; 2 disulfide linkages are also predicted (Generated from PDBsum).

The model generated by MODELLER was subject to energy minimization and assessed for both geometric and energy aspects using Swiss-Pdb Viewer and refined using What If Web Interface. The final model (Figure 1) showed a quality factor of 83.143% in ERRAT. The positioning of secondary structural elements was generated from PDBsum. In all, the predicted model of *bu*PAG2 was found to contain 7 sheets, 9 beta hairpins, 2 psi loops, 6 beta bulges, 26 strands, 15 helices, 4 helix-helix interactions, 32 beta turns, 6 gamma turns and 2 disulphide linkages (Figure 2).

**Figure 3:**
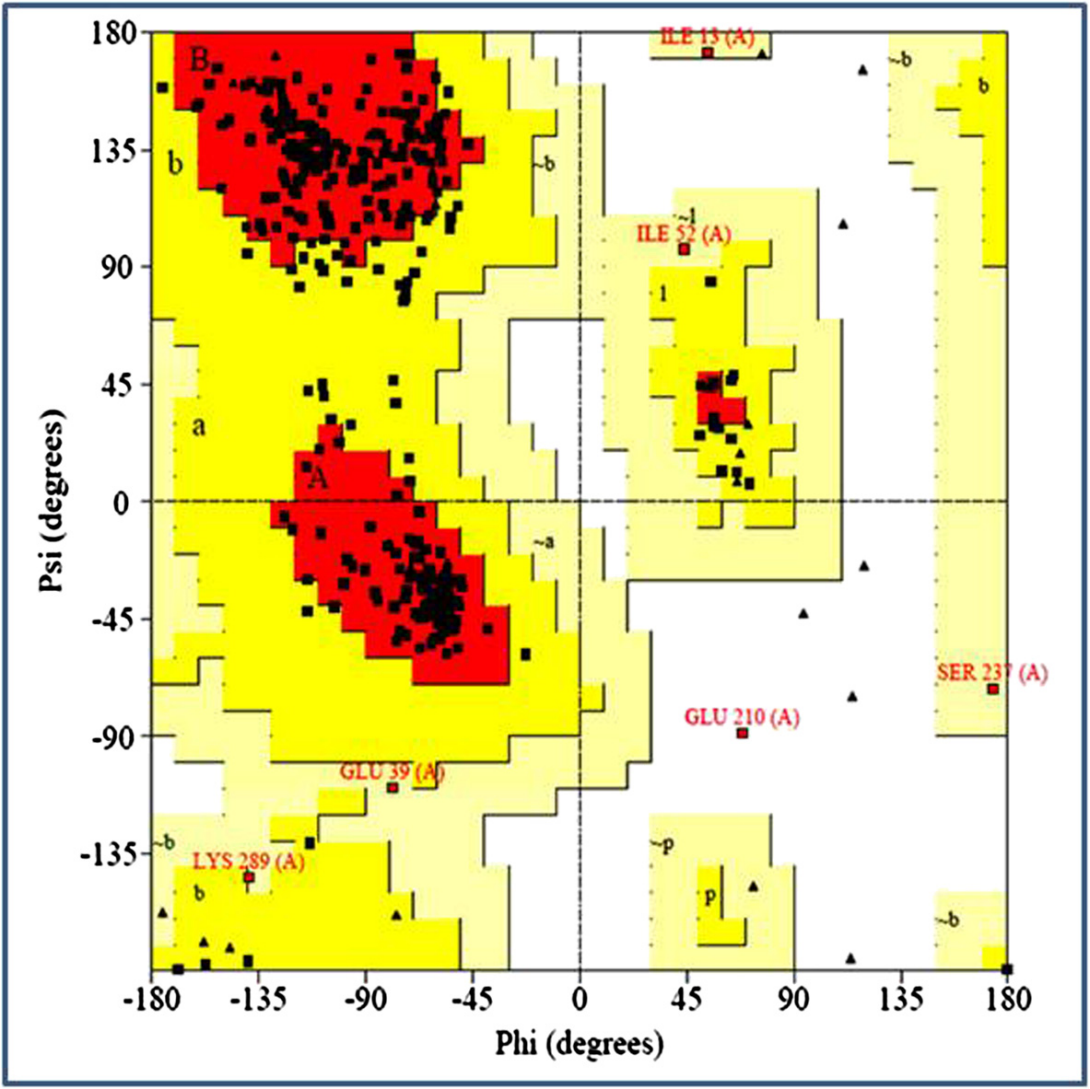
**Ramachandran plot for the predicted model of*bu*PAG2.** 1 outlier (Glu 210) is present. 5 residues (Ile 13, Glu 39, Ile 52, Ser 237 and Lys 289) are present in the generously allowed region. All other 361 residues are in the allowed regions (Generated from PDBsum).

Several structure assessment methods including Ramachandran plots and RMSD were used to check the reliability of the predicted 3D model. Ramachandran plots were also obtained from PDBsum for quality assessment. Only 1 (0.3%) of the total 367 residues were present in the disallowed region whereas another 5 residues were present in the generously allowed regions (Figure 3). G-factors provide a measure of how unusual a stereo-chemical property is. Values below −0.5 represent unusual property where as, values below −1.0 represent high unusualness. The G-factors for dihedral angles and main chain covalent forces were calculated to be −0.37 and 0.14, respectively. The overall average G-factor for the *bu*PAG2 model was −0.16. The Ramachandran plot and G-factors indicate that the backbone dihedral angles, phi and psi, in the 3D model of *bu*PAG2 are well within acceptable limits.

**Figure 4:**
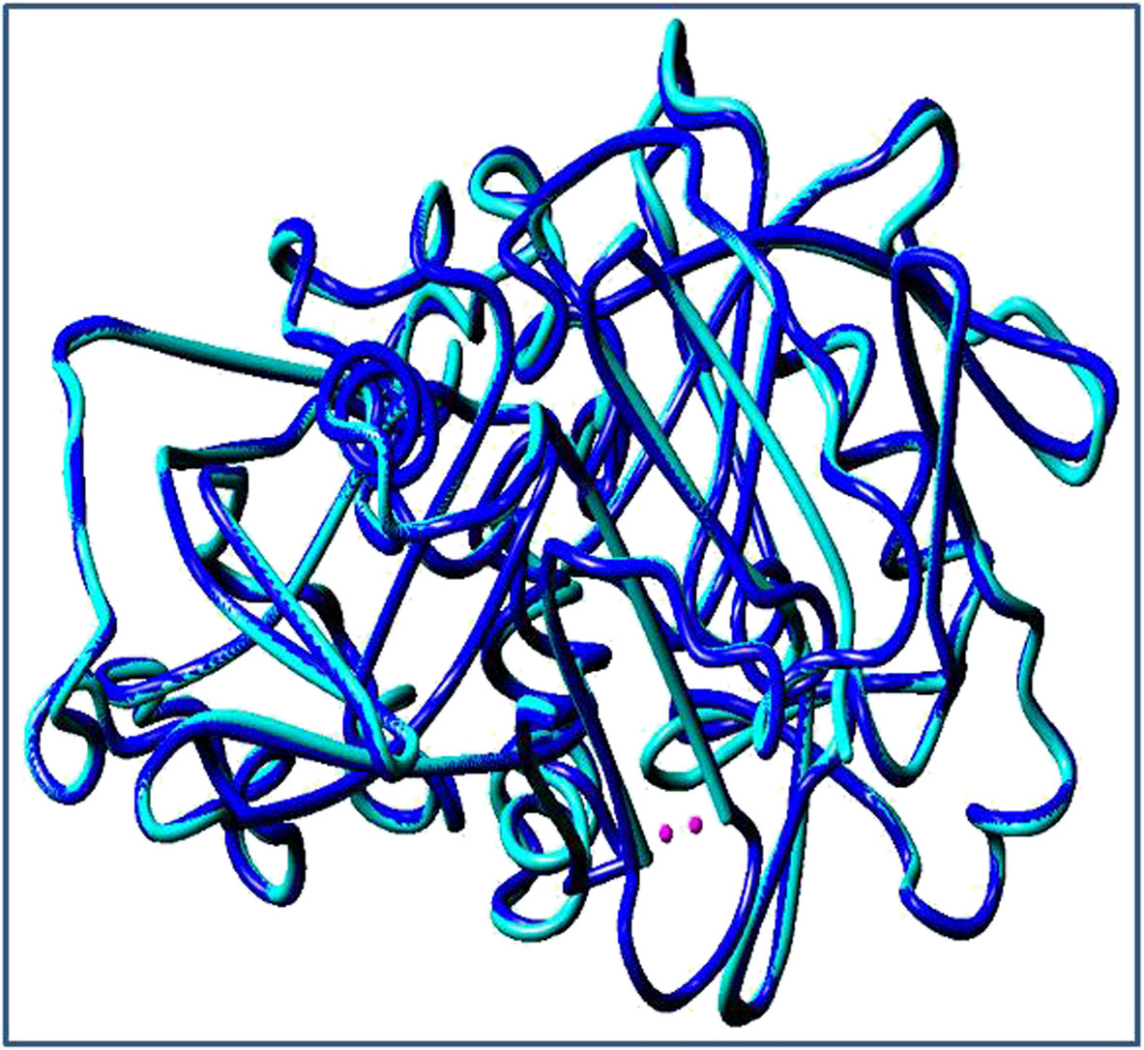
**Structural alignment of*bu*PAG2 with the template 3PSG_A.** Structural alignment of predicted model of *bu*PAG2 (blue) with the template 3PSG_A (cyan) is shown. RMSD value of 0.447 Å was found over 353 aligned residues (Calculated with MUSTANG implementation in YASARA View).

The Root Mean Square Deviation (RMSD) indicates the degree to which two 3D structures are similar; the lower the value, the more similar the structures. Both template and query structures were superimposed for the calculation of RMSD (Figure 4). The RMSD value obtained from superimposition of *bu*PAG2 and 3PSG_A, using MUSTANG in YASARA View, was found to be 0.447 Å over a total of 353 aligned residues. The overall quality factor, Ramachandran plot characteristics, G-factors and RMSD values confirm the quality of the homology model of *bu*PAG2. The final protein structure was deposited in PMDB [PMDB: PM0077895].

**Figure 5:**
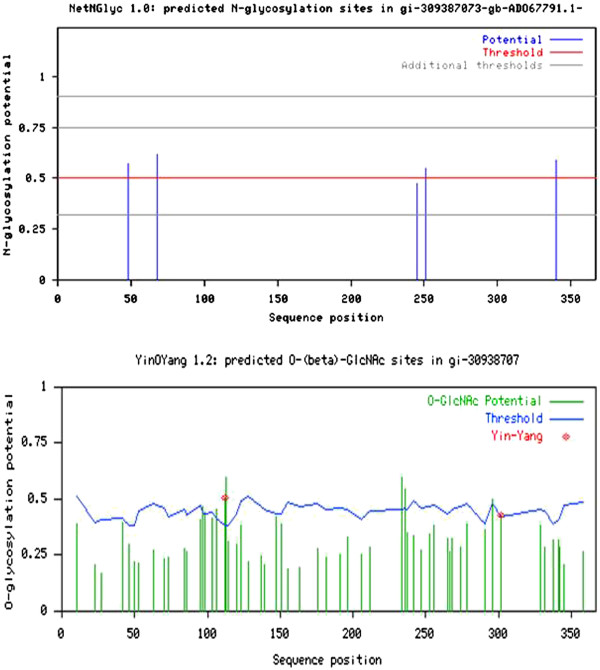
**Prediction of glycosylation sites in*bu*PAG2. a.** 4 N-glycosylation sites were predicted by NetNGlyc 1.0. **b.** 5 O-(beta)-GlcNac sites and 2 Yin-Yang sites were predicted by YinOYang 1.2. No O-glycosylation sites were predicted.

**Figure 6:**
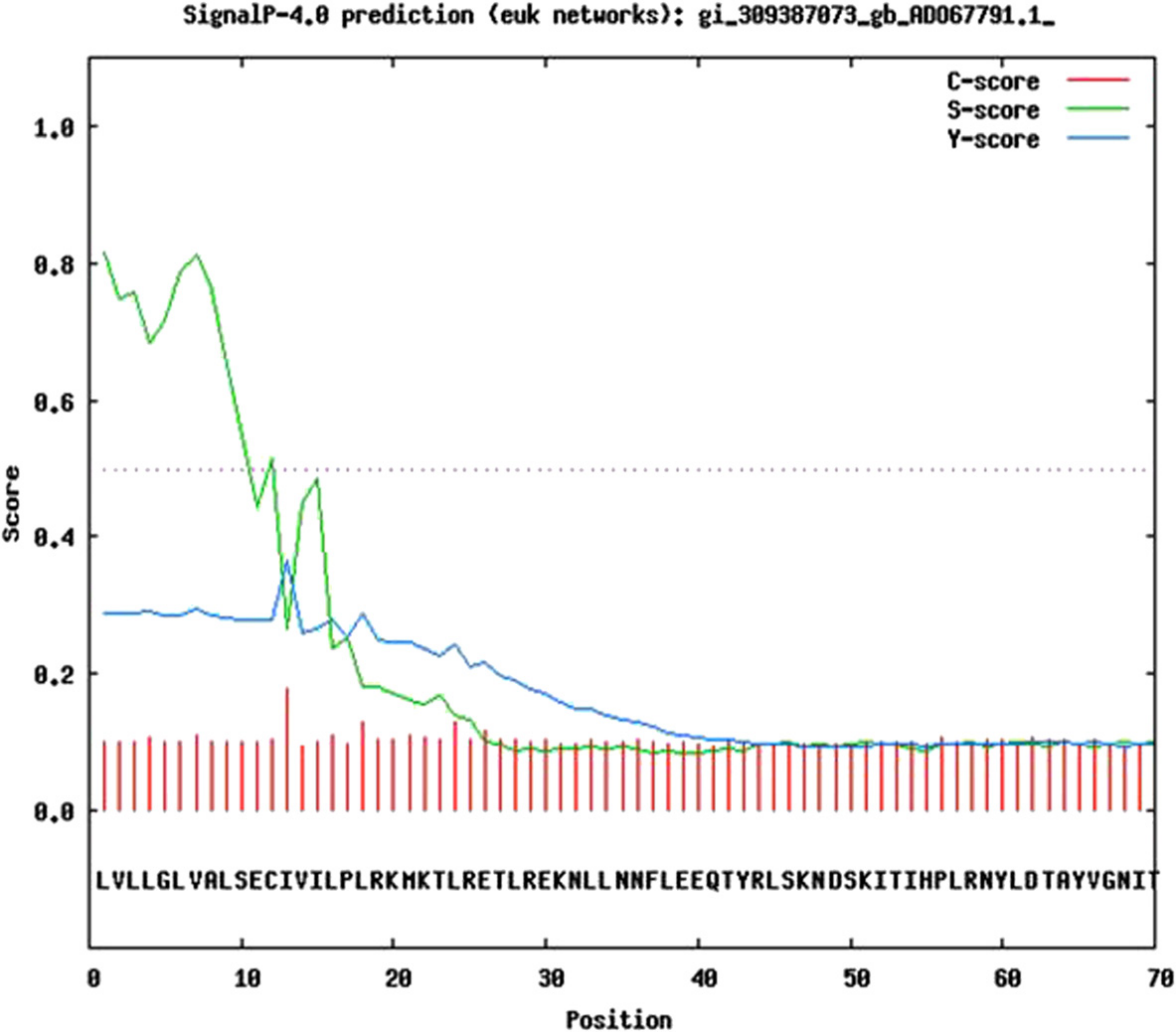
**Prediction of signal peptide in buPAG2.** Residues 1–12 are predicted as the signal peptide and cleavage site is predicted between residues 12 and 13 by SignalP 4.0. Residues 13–41 were predicted by Pfam as the propeptide.

The glycosylation sites were predicted by using NetOGlyc, NetNGlyc and YinOYang tools provided by CBS DTU (Figure 5). NetOGlyc could not detect any O-glycosylation sites; NetNGlyc predicted N-glycosylation sites at residues 48, 68, 251 and 340. One N-glycosylation was also predicted with low confidence at position 245. YinOYang predicted 5 O-(beta)-GlcNAc sites at residues 112, 113, 234, 236 and 296. Four other sites were also predicted with low confidence at residues 97, 98, 106 and 302. Of the total 9 sites, residues 112 and 302 were also predicted as Yin-Yang sites. Yin-Yang sites are Ser/ Thr residues that are O-(beta)-GlcNAcylated as well as phosphorylated; these are reversibly and dynamically modified by O-GlcNAc or Phosphate groups at different times. Butler et al. also recorded large disparity in the glycosylation pattern of PAGs [1]. SignalP recognized the first 12 residues in the sequence as a signal peptide for extracellular secretion of the protein (Figure 6).

### Functional annotation of buPAG2

Presently, PAGs are known to be pregnancy induced proteins expressed about 7^th^ day post-fertilization onwards largely in the pre-placental trophoblast, and post-implantation trophectoderm. In the present study, a systematic workflow consisting of several bioinformatics tools and databases was defined and used with the goal of performing structural and functional annotation of *bu*PAG2. Three web tools were used to search the conserved domains and potential function of *bu*PAG2. Based on consensus predictions made by Pfam, NCBI-CDD and InterProScan, it is confirmed that buPAG2 belongs to the aspartate protease superfamily and possesses eukaryotic aspartyl protease domain. Aspartic proteases are a family of protease enzymes that use an aspartate residue for catalysis of their peptide substrates. In general, they have two highly-conserved aspartates in the active site and are optimally active at acidic pH.

Eukaryotic aspartic proteases include pepsins, cathepsins, and renins. They have a two-domain structure, arising from ancestral duplication. Each domain contributes a catalytic Asp residue, with an extended active site cleft localized between the two lobes of the molecule. One lobe has probably evolved from the other through a gene duplication event in the distant past. In modern-day enzymes, although the three-dimensional structures are very similar, the amino acid sequences are more divergent, except for the catalytic site motif, which is much conserved. The presence and position of disulfide bridges are other conserved features of aspartic peptidases [26,27].

Most eukaryotic endopeptidases are synthesized with signal and propeptides. The animal pepsin-like endopeptidase propeptides form a distinct family of propeptides, which contain a conserved motif approximately 30 residues long. The propeptide contains two helices that block the active site cleft; in particular, the conserved Asp residue in the protease hydrogen bonds to a conserved Arg residue in the propeptide. This hydrogen bond stabilizes the propeptide conformation and is probably responsible for triggering the conversion of the zymogen to active enzyme under acidic conditions [26,27].

In our structure, Pfam recognized a 29 amino-acid long, propeptide sequence from residues 13 to 41 and a 304 amino-acid long, eukaryotic aspartyl protease sequence from 64 to 367 residues. Active sites of the protease were recognized at positions 83 and 264. The first 12 residues were recognized by SignalP as the signal peptide. The propeptide was recognized as a member of the A1 Propeptide family (PF07966), whereas the aspartyl protease was recognized as a member of the Asp family (PF00026) under the peptidase clan AA (CL0129). These predictions are similar to those of InterProScan that recognized peptidase activity within residues ranging from 71–91, 212–225, 261–272 and 346–361. The catalytic sites for the protease were predicted by InterProScan within residue range from 54–219 and 225–367; active sites were predicted to be present within residue range from 80–91 and 261–272. The peptidase clan AA (CL0129) contains aspartic peptidases, including the pepsins and retropepsins. These enzymes contain a catalytic dyad composed of two aspartates. In the retropepsins one is provided by each copy of a homodimeric protein, whereas in the pepsin-like peptidases these aspartates come from a single protein composed of two duplicated domains. This clan contains the 12 member families, *viz*. Asp, Asp protease, Asp protease 2, DUF1758, gag-asp protease, Peptidase A2B, Peptidase A2E, Peptidase A3, RVP, RVP 2, Spuma A9PTase and Zn protease [26-28].

NCBI-CDD could also recognize A1 Propeptide (cl06833); and cellular and retroviral pepsin-like protease (cl11403) superfamily sequences within *bu*PAG2. This superfamily is further classified as the peptidase family A1 (pepsin A) and A2 (retropepsin family). Specifically, the alignment of *bu*PAG2was detected with the superfamily member cd05478, *i.e.* Pepsin A. The cellular pepsin and pepsin-like enzymes are twice as long as their retroviral counterparts. These are found in mammals, plants, fungi and bacteria. These well known and extensively characterized enzymes include pepsins, chymosin, rennin, cathepsins, and fungal aspartic proteases. They contain two domains possessing similar topological features. The N- and C-terminal domains, although structurally related by a 2-fold axis, have only limited sequence homology except in the vicinity of the active site, suggesting that the enzymes evolved by an ancient duplication event. The eukaryotic pepsin-like proteases have two active site Asp residues with each N- and C-terminal lobe contributing one residue. While the fungal and mammalian pepsins are bilobal proteins, retropepsins function as dimers and the monomer resembles structure of the N- or C-terminal domains of eukaryotic enzyme. The active site motif (Asp-Thr/Ser-Gly-Ser) is conserved between the retroviral and eukaryotic proteases and between the N-and C-terminal of eukaryotic pepsin-like proteases. These endopeptidases specifically cleave bonds in peptides at least six residues in length with hydrophobic residues in both the P1 and P1' positions. The active site is located at the groove formed by the two lobes, with an extended loop projecting over the cleft to form an 11-residue flap, which encloses substrates and inhibitors in the active site. Specificity is determined by nearest-neighbor hydrophobic residues surrounding the catalytic aspartates, and by three residues in the flap. Nearly all known aspartyl proteases are inhibited by pepstatin [26-28]. In our model, the inhibitor binding site was predicted by NCBI-CDD to be formed of residues 83, 85, 87, 123, 124, 125 and 169. NCBI-CDD could predict only one active site at residue 83 within a catalytic motif formed by residues 83–85. Additionally, active site flaps were predicted at residues 123–126 and 130–133.

ProKnow metaserver integrates outputs from PSI-BLAST, PROSITE, DALI/ DASEY, DIP and RIGOR to extract similarity of the query sequence with proteins in the ProKnow database. This information is subsequently used to assign a weighted set of functions to the query protein. Consensus results from ProKnow and PFP servers suggest inhibitory effects of *bu*PAG2 on proteolysis, immunological response and carbohydrate metabolism. *Bu*PAG2 shows strong evidence for MHC I binding and down-regulation of the complement pathway. Hashizume and co-workers put forth that PAGs may act as immunosuppressants allowing for the immunological acceptance of the embryo by the dam [4]; such effects may be accounted for in part by MHC binding and complement inhibiting activity of PAGs. Also, a role of *bu*PAG2 in regulation of transcription is predicted at moderate confidence level, possibly through DNA dependent and GTP binding mechanisms. Pregnancy is a complex physiological process requiring adaptations by the dam on many fronts. While down-regulation of immune response is deemed essential for acceptance of the fetus as a hemi-allograft, down-regulation of proteolysis and carbohydrate metabolism may have nutritional consequences. Alternately, down-regulation of proteolysis may also be an essential pre-requisite for controlled apoptotic processes underlying fetal morphogenesis and/ or re-modeling of feto-maternal tissues; similar roles for PAGs have been postulated in bovines by Hashizume et al. [4]. Similarly, regulation of transcription may also be required for orchestration of a multitude of physiological processes in response to pregnancy. PFP also recognizes *bu*PAG2 as an inducible, extracellular protein. Successful maintenance and consummation of pregnancy requires the dam to produce molecular signals, mainly proteins, which are involved in vital processes as blockage of PGF2α secretion and endometrial remodeling [29,30]. A role of PAGs in implantation and placentogenesis has also been proposed by Ishiwata et al. [31]. PAGs have also been shown to possess luteotropic activity [32,33].

BlastP against microbial and eukaryotic DEG entries did not recognize *bu*PAG2 or an ortholog as a gene product that is essential for survival of an organism. Based on a KEGG search performed *via* KAAS, again, *bu*PAG2 was not found to be essentially involved in any of the biometabolic pathways. The essentiality of an inducible gene product with sex-restricted expression in pregnant females is logically unlikely.

## Conclusion

In this study, homology modeling and comparative genomics approach has been used to propose the first 3D structure and possible functions for *bubaline* Pregnancy associated glycoprotein 2. With the assistance of a well-defined structure and annotations, the functional and binding sites have been predicted, which will further the understanding of the biological roles of the protein. Our study predicts *bu*PAG2 as an inducible, extra-cellular, non-essential, N-glycosylated, aspartic pro-endopeptidase that is involved in down-regulation of complement pathway and immunity during pregnancy. The protein is also predicted to be involved in such down-regulation of proteolysis and carbohydrate metabolism, and regulation of transcription, as may be an essential pre-requisite for controlled apoptotic processes underlying fetal morphogenesis and re-modeling of feto-maternal tissues. These structural and functional insights shall allow the designing of recombinant, lack-of-function proteins, and inhibitors for dissection of the exact physiological role of the PAGs.

## Competing interests

The authors declare that they have no competing interests.

## Authors’ contributions

BG carried out sequence retrieval, homology modeling, model optimization, structural-functional annotation and drafted the manuscript. SP conceived the study, and participated in its design and coordination and helped to draft the manuscript. Both authors read and approved the final manuscript.

## References

[B1] Butler JE Hamilton WC Sasser RG Ruder CA Hass GM Williams RJ Detection and partial characterization of two bovine pregnancy-specific proteins Biol Reprod 1982 26 925 933 10.1095/biolreprod26.5.925 6807365

[B2] Zoli AP Beckers JF Wouters-Ballman P Closset J Falmagne P Ectors F Purification and characterization of a bovine pregnancy-associated glycoprotein Biol Reprod 1991 45 1 10 10.1095/biolreprod45.1.1 1908709

[B3] Garbayo JM Green JA Mannikam M Beckers JF Kiesling DO Early AD Roberts M Caprine pregnancy associated glycoproteins (PAG): their cloning, expression and evolutionary relationship to other PAG Mol Reprod Dev 2000 57 311 322 10.1002/1098-2795(200012)57:4<311::AID-MRD2>3.0.CO;2-F 11066059

[B4] Hashizume K Ushizawa K Patel OV Kizaki K Imai K Yamada O Nakano H Takahashi T Gene expression and maintenance of pregnancy in bovine roles of trophoblastic binucleate cell specific molecules Reprod Fert Dev 2007 19 79 90 10.1071/RD06118 17389137

[B5] Benson DA Karsch-Mizrachi I Lipman DJ Ostell J Wheeler DL GenBank Nucleic Acids Res 2007 35 21 25 17202161 10.1093/nar/gkl986 PMC1781245

[B6] Gasteiger E Hoogland C Gattiker A Duvaud S Wilkins MR Appel RD 2005 Protein Identification and Analysis Tools on the ExPASy Server. In The Proteomics Protocols Handbook. Edited by Walker JM. Humana Press, Bairoch A 571 607

[B7] Altschul SF Madden TL Schaeffer AA Zhang J Zhang Z Miller W Lipman DJ Gapped BLAST and PSI-BLAST: a new generation of protein database search programs Nucleic Acids Res 1997 25 3389 3402 10.1093/nar/25.17.3389 9254694 PMC146917

[B8] Sali A Potterton L Yuan F van Vlijmen H Karplus M Evaluation of comparative protein modeling by MODELLER Proteins 1995 23 318 326 10.1002/prot.340230306 8710825

[B9] Guex N Peitsch MC SWISS-MODEL and the Swiss-PdbViewer: An environment for comparative protein modelling Electrophoresis 1997 18 2714 2723 10.1002/elps.1150181505 9504803

[B10] van Gunsteren WF Biomolecular Simulation: The GROMOS96 Manual and User Guide 1996 Vdf Hochschulverlag ETHZ, 1 1042

[B11] Vriend G WHAT IF: A molecular modeling and drug design program J Mol Graph 1990 8 2 56 10.1016/0263-7855(90)80062-K 2268628

[B12] Laskowski RA Hutchinson EG Michie AD Wallace AC Jones ML Thornton JM PDBsum: a Web-based database of summaries and analyses of all PDB structures Trends Biochem Sci 1997 22 488 490 10.1016/S0968-0004(97)01140-7 9433130

[B13] Colovos C Yeates TO Verification of protein structures: patterns of non bonded atomic interactions Protein Sci 1993 2 1511 1519 10.1002/pro.5560020916 8401235 PMC2142462

[B14] Krieger E Koraimann G Vriend G Increasing the precision of comparative models with YASARA NOVA - a self-parameterizing force field Proteins 2002 47 393 402 10.1002/prot.10104 11948792

[B15] Konagurthu AS Whisstock JC Stuckey PJ Lesk AM MUSTANG: A multiple structural alignment algorithm Proteins 2006 64 559 574 10.1002/prot.20921 16736488

[B16] Gupta R Brunak S Prediction of glycosylation across the human proteome and the correlation to protein function Pacific Symposium on Biocomputing 2002 7 310 322 11928486

[B17] Petersen TN Brunak S von Heijne G Nielsen H SignalP 4.0: Discriminating signal peptides from transmembrane regions Nature Methods 2011 8 785 786 10.1038/nmeth.1701 21959131

[B18] Castrignanò T De Meo PD Cozzetto D Talamo IG Tramontano A The PMDB Protein Model Database Nucleic Acids Res 2006 34 Database issue D306 D309 16381873 10.1093/nar/gkj105 PMC1347467

[B19] Zdobnov EM Apweiler R InterProScan - an integration platform for the signature-recognition methods in InterPro Bioinformatics 2001 17 847 848 10.1093/bioinformatics/17.9.847 11590104

[B20] Finn RD Mistry J Tate J Coggill P Heger A Pollington JE Gavin OL Gunasekaran P Ceric G Forslund K Holm L Sonnhammer EL Eddy SR Bateman A The Pfam protein families database Nucleic Acids Res 2010 38 Database issue D211 D222 19920124 10.1093/nar/gkp985 PMC2808889

[B21] Marchler-Bauer A Lu S Anderson JB Chitsaz F Derbyshire MK DeWeese-Scott C Fong JH Geer LY Geer RC Gonzales NR Gwadz M Hurwitz DI Jackson JD Ke Z Lanczycki CJ Lu F Marchler GH Mullokandov M Omelchenko MV Robertson CL Song JS Thanki N Yamashita RA Zhang D Zhang N Zheng C Bryant SH CDD: a Conserved Domain Database for the functional annotation of proteins Nucleic Acids Res 2011 39 Database issue D225 D229 21109532 10.1093/nar/gkq1189 PMC3013737

[B22] Pal D Eisenberg D Inference of protein function from protein structure Structure 2005 13 121 130 10.1016/j.str.2004.10.015 15642267

[B23] Hawkins T Luban S Kihara D Enhanced automated function prediction using distantly related sequences and contextual association by PFP Protein Sci 2006 15 1550 1556 10.1110/ps.062153506 16672240 PMC2242549

[B24] Zhang R Ou HY Zhang CT DEG: a database of essential genes Nucleic Acids Res 2004 32 Database issue D271 D272 14681410 10.1093/nar/gkh024 PMC308758

[B25] Moriya Y Itoh M Okuda S Yoshizawa AC Kanehisa M KAAS: an automatic genome annotation and pathway reconstruction server Nucleic Acids Res 2007 35 Web Server issue W182 W185 17526522 10.1093/nar/gkm321 PMC1933193

[B26] Rawlings ND Barrett AJ Evolutionary families of peptidases Biochem J 1993 290 205 218 8439290 10.1042/bj2900205 PMC1132403

[B27] Cooper JB Khan G Taylor G Tickle IJ Blundell TL X-ray analyses of aspartic proteinases. II. Three-dimensional structure of the hexagonal crystal form of porcine pepsin at 2.3 Å resolution J Mol Biol 1990 214 199 222 10.1016/0022-2836(90)90156-G 2115088

[B28] Rawlings ND Morton FR Kok CY Kong J Barrett AJ MEROPS: the peptidase database Nucleic Acids Res 2008 36 D320 D325 10.1093/nar/gkn292 17991683 PMC2238837

[B29] Sousa NM Ayad A Beckers JF Gajewski Z Pregnancy associated glycoproteins (PAG) as pregnancy markers in the ruminants J Phy Pharm 2006 57 supp 8 153 171 17242480

[B30] Spencer TE Johnson GA Bazer FW Burghardt RC Implantation mechanisms: insights from the sheep Reproduction 2004 128 657 668 10.1530/rep.1.00398 15579583

[B31] Ishiwata H Katsuma S Kizaki S Patel OV Nakano H Takahashi T Imai K Hirasawa A Shiojima S Ikawa H Suzuki Y Tsujimoto G Izaike Y Todoroki J Hashizume K Characterization of gene expression profiles in early bovine pregnancy using a custom cDNA microarray Mol Reprod Dev 2003 65 9 18 10.1002/mrd.10292 12658628

[B32] Beckers JF Roberts RM Zoli AP Ectors F Derivaux J Molecules of the family of aspartic proteinases in the placenta of ruminants: hormones or proteins? Bull Mem Acad R Med Belg 1994 149 355 367 7550037

[B33] Weems CW Weems YS Randel RD Prostaglandin and reproduction in female farm animals Vet J 2006 171 206 228 10.1016/j.tvjl.2004.11.014 16490704

